# Towards Semantic Smart Cities: A Study on the Conceptualization and Implementation of Semantic Context Inference Systems

**DOI:** 10.3390/s23239392

**Published:** 2023-11-24

**Authors:** Jieun Lee, JaeSeung Song

**Affiliations:** Depatment of Convergence Engineering for Intelligent Drone, Sejong University, Gwangjin-gu, Seoul 05006, Republic of Korea; love9ly@sju.ac.kr

**Keywords:** semantics, smart city, context extraction, semantic reasoning

## Abstract

Smart cities provide integrated management and operation of urban data emerging within a city, supplying the infrastructure for smart city services and resolving various urban challenges. Nevertheless, cities continue to grapple with substantial issues, such as contagious diseases and terrorism, that pose severe financial and human risks. These problems sporadically arise in various locales, and current smart city frameworks lack the capability to autonomously identify and address these issues. The challenge intensifies especially when trying to recognize and respond to unprecedented problems. The primary objective of this research is to predict potential urban issues and support their resolution proactively. To achieve this, our system makes use of semantic reasoning to understand the ongoing situations within the city. In this process, the 5W1H principles serve as inference rules, guiding the extraction and consolidation of context. Firstly, utilizing domain-specific annotation templates, we craft a semantic graph by amalgamating information from various sources available in the city, such as municipal public data and IoT platforms. Subsequently, the system autonomously infers and accumulates contexts of situations occurring in the city using 5W1H-based reasoning. As a result, the accumulated contexts allow for inferring potential urban problems by identifying repeated disruptions in city services at specific times or locations and establishing connections among them. The main contribution of this paper lies in proposing a comprehensive conceptual model for the suggested system and presenting actual implementation cases and applicable use cases. These contributions facilitate awareness among city administrators and citizens within a smart city regarding potential problem-prone areas or times, thereby aiding in the preemptive identification and mitigation of urban challenges.

## 1. Introduction

According to a 2018 survey by the United Nations, 55% of the world’s population resides in urban areas. This urban concentration precipitates various challenges in fields such as habitation, transportation, and the urban environment. The term urban problems refers to issues that arise due to city population density and urbanization, such as unemployment and traffic congestion during commuting hours. A smart city refers to an urban area that offers services aimed at resolving these issues through the comprehensive management of data collected from sensors installed throughout the city [1]. Based on an open IoT platform, a smart city secures real-time data and supports smooth data flow, such as traffic volume and a wide array of other substantial data. This enables services such as air pollution measurement to tackle environmental pollution issues, providing information about air quality, and measuring and predicting traffic volume to address traffic congestion issues [2,3].

However, for the establishment of smart cities to result in the resolution of urban issues, it is crucial, first and foremost, to perceive urban situations in real time and to comprehend or predict in advance the causes of the problems emerging within cities. There are persistent challenges in urban areas such as infectious diseases, housing shortages, and unemployment, yet smart cities to date encounter difficulties in autonomously recognizing these urban problems [4]. Specifically, issues like chronic congestion and a decrease in subway ridership during commute hours persistently occur in major cities and various sections, but pinpointing the exact times, locations, and causes of these problems is not straightforward.

This difficulty is primarily due to the characteristics of smart cities that provide various industry services, including parking management and energy management, across different fields. In most cases, the causes of urban problems that occur in various parts of the city cannot be attributed to only a single factor due to the diverse services offered by smart cities. Such urban problems generally emerge from a complex overlay of multiple situations. For example, even when analyzing a single traffic accident that occurred within a city, various factors, such as the driver’s driving habits, the size of the intersection, and the speed of the vehicle, all serve as contributing causes to the accident, leading to variations in the severity of injuries and the extent of damage [5].

A context-awareness system utilizing semantic technology can offer a solution to the difficulty of identifying the causes of urban problems in smart cities [6]. Semantic technology establishes logical relationships between data, allowing for the identification of places or times where urban problems frequently occur based on the city’s characteristics, thereby connecting all related data. Data linked in this manner can be organically connected through a context-awareness system without being confined to a specific field or domain, aiding in the identification of various causes of urban problems. Specifically, the development of technology that can infer potential risks by considering various urban situations comprehensively is necessary in order to provide specialized cause analysis and intelligent predictive services for urban problems in smart cities [7,8].

In this study, we first extracted the requirements and conceptual model of a Semantic Context Inference System (SeCIS) which can deduce urban situations as contexts and predict urban problems through logical reasoning. The proposed SeCIS is composed of a four-layer architecture: data access layer, semantic acquisition layer, context extraction layer, and inference application layer. The data access layer collects smart city data, which describe factors influencing urban problems from various sources such as IoT platforms and public data portals, and unifies the format of diverse data sets based on smart city data schemas for reusability. In the semantic acquisition layer, a semantic graph is constructed based on smart city ontology, and contexts are deduced in the context extraction layer using the 5W1H principles. Our prediction approach accumulates contexts that are connected as a single context considering factors like time, place, and weather. When SeCIS recognizes a service disruption in the city, it infers events causing urban problems by connecting contexts based on location and time. As a result, the inference application layer can preemptively warn administrators or individual city dwellers of potential urban problems or service disruptions when events causing these problems are detected at the same time and place. Moreover, feedback on the causal relationship between events and urban problems from city administrators is collected and circulated back to the context extraction layer, thereby establishing continually evolving rules for logical reasoning.

In summary, our main contributions are as follows:

- Definition of the conceptual model and layer-specific requirements of the Semantic Context Inference System for an urban problem.
- An approach that performs real-time semantic annotation for the relationships between smart city data using a smart city ontology.
- A mechanism for extracting semantic context for describing the current urban situation.

The rest of this paper is organized as follows. In Section 2, we study the related technologies and extract the requirements for the four layers. Section 3 presents the conceptual model and composition of the Semantic Context Inference System (SeCIS). In Section 4, we describe the methodology and implementation used to estimate urban problem inferences and introduce use cases that employ this system. Section 5 details the validation of the SeCIS implementation, demonstrating the system’s effectiveness within a controlled setting. Finally, Section 6 concludes the paper and discusses future work.

## 2. Previous Research and Requirements

In this section, a system is proposed that extracts contextual knowledge from data collected in smart cities, enabling the recognition of various situations occurring within the city. This section aims to examine, from a comprehensive perspective, an overview of the contextual knowledge that can be inferred from smart cities and the component technologies of semantic context inference, categorized by field (data access, semantic acquisition, context extraction, inference application). Additionally, requirements for the Semantic Context Inference System (SeCIS) are identified based on existing literature related to semantic technologies.

### 2.1. Overview of Semantic Knowledge and Context

While syntactic knowledge encompasses the way words are assembled and sentences are structured in a particular language, semantic knowledge involves the meaning found in the actual text, symbols, and signs, with a focus on understanding the situations and contexts in urban environments. In comparison with humans, contextual knowledge refers to new knowledge and features that constitute concepts that individuals have acquired and abstracted from their experiences [9]. Traditional smart city systems can collect data and generate statistics, but they are incapable of classifying or analyzing data to discern their contextual meaning and derive new insights.

Conversely, semantic technology enables not only the understanding of sentence structures and word meanings but also the derivation of semantic data, such as concepts, features, and domains. Furthermore, it allows for the derivation of new concepts and knowledge [10]. Especially in smart city environments, where massive numbers of data from various domains are generated, the advantages of semantic technology are more pronounced. However, despite these advantages, semantic technology remains underutilized due to the high barrier of requisite background knowledge needed for understanding it, as well as the complexity involved in defining inference rules to meet the information needs of the intended system.

Typically, citizens or administrators in a smart city assume that their information needs can be satisfied since large volumes of data are being collected. However, in many cases, it is challenging to realize this assumption in practice. While it is possible to provide information that citizens wish to know in some cases, like weather and air quality, it is often difficult for them to explicitly articulate their information needs in most instances. Particularly for recognizing current phenomena or situations occurring in a smart city, the system should be structured to reflect citizens’ information needs and incorporate clear inference rules for this purpose.

### 2.2. Research on Key Technologies by Layer

Syntactic knowledge extraction necessitates understanding not only the relationships between domains but also the ability to perform logical inference based on the complex connection information between data to provide satisfactory knowledge extraction results. However, there are limitations to fragmented approaches through simplistic conceptual architectures like statistics or analysis of the existing data. Therefore, in this section, we will explore related research from a comprehensive perspective, dividing the key technologies of semantic context inference into four layers: data access layer, semantic acquisition layer, context extraction layer, and inference application layer. Through these four layers, we can extract semantic information from data, transform it into high-level knowledge, and ultimately apply this contextual knowledge to real use cases.

#### 2.2.1. Data Access

Efforts are being made in smart cities to efficiently operate the cities and enhance the quality of life for their residents by adopting and utilizing big data platforms for data access [11]. In this context, technology that performs data collection from various urban infrastructures is pivotal, serving to systematically manage and utilize the diverse and vast data generated within a city [12]. Research on distributed processing systems like Apache Hadoop is ongoing to facilitate this need.

For instance, South Korea’s smart city data platform technology, known as City Data Hub, exemplifies one of these efforts. The City Data Hub collects raw data through international standard protocols and standard interfaces from various city systems, including IoT platforms, administrative systems, integrated smart city platforms, and legacy systems. This approach enables the integrated management of the collected data [13].

Specifically, it employs protocols such as MQTT and CoAP for real-time data communication between IoT devices and interfaces that adhere to OpenAPI specifications for seamless integration between different systems. Furthermore, for data format and exchange, it utilizes JSON and XML, which are widely recognized and accepted standards in the industry. To store vast numbers of data, one can utilize NoSQL databases like MongoDB and Cassandra, relational databases, or Big Data processing tools such as Apache Hadoop and Apache Spark.

However, the utilization of NoSQL databases and Big Data processing tools like Apache Spark also brings forth challenges and limitations. NoSQL databases, while providing scalability and flexibility, may pose issues regarding data consistency and transactional integrity, which are crucial in managing critical urban infrastructure data reliably. Additionally, these databases require specialized skills and knowledge to manage, which might escalate the operational complexity and learning curve for the administrators. Big Data processing tools, on the other hand, necessitate substantial computational resources and expertise for optimization and maintenance, posing a challenge in environments where these resources are limited or costly. Also, ensuring data privacy and security while processing and analyzing large datasets is a constant challenge, given the sensitive nature of urban data collected in smart cities.

#### 2.2.2. Semantic Acquisition

Ontologies, pivotal in semantic technologies, establish structured frameworks for information representation. They adopt a triple-form structure (subject, predicate, object) that enables a more intuitive and accessible semantic processing of data. Semantic acquisition’s primary goal is to convert disparate content sources—including text, images, and multimedia—into semantic metadata informed by ontological structures.

Fundamental technologies in the perspective of semantic information acquisition include natural language processing (NLP), statistical methods, and machine learning techniques. Libraries for NLP such as SpaCy, NLTK, and Gensim; information retrieval tools like Apache Lucene and Elasticsearch; and machine learning libraries such as scikit-learn, TensorFlow, and PyTorch can all be utilized in the process of semantic acquisition.

On the other hand, prior research has demonstrated varied ontology-based approaches to semantic technology applications [14,15]. For example, ontologies have been leveraged in urban planning, healthcare, and environmental monitoring, showcasing their versatility across different domains. In urban environments, semantic ontologies have played a significant role in integrating heterogeneous data sources, which has been crucial for the development of smart cities. Studies have shown how ontologies can be employed to consolidate information across transportation, utility, and emergency response systems to facilitate better urban management [16,17].

The benefits of using ontological models include the enhanced ability to standardize and link data, thus providing a more cohesive view of complex systems [18,19]. For instance, the adoption of the SSN ontology (semantic sensor network) in environmental monitoring projects has yielded improved data interoperability and analysis capabilities, enabling better-informed decision making processes.

However, limitations also emerge, primarily related to the complexity of ontology development and maintenance. The difficulty of capturing the nuanced and evolving nature of real-world relationships in a static ontological model presents a constant challenge [20,21]. Moreover, the extensive effort required to annotate data manually for ontology-based systems is a limitation that ongoing research aims to address through automation [22].

Illustrative case studies offer concrete insights into the practical application of these technologies. In Barcelona’s smart city initiative, semantic technologies facilitated data integration from diverse domains such as public transportation, energy consumption, and citizen services, thereby enhancing urban planning and operational efficiency [23]. Similarly, Singapore’s semantic traffic management system illustrates how machine learning, intertwined with ontology-driven analytics, can enhance real-time traffic flow management, reducing congestion and improving urban mobility [24].

Reflecting on the feedback received, this section could be improved by providing a more comprehensive analysis of how ontologies are specifically tailored for various urban challenges. Additionally, future research should focus on the ontological engineering process, detailing the selection, customization, and validation of ontologies within the context of semantic acquisition. Competency questions that define the scope and requirements of ontological models could further refine the process and ensure alignment with the targeted outcomes.

#### 2.2.3. Context Extraction

Context extraction plays an essential role in transforming raw, unstructured data into meaningful, actionable insights within smart cities by analyzing the context in which data are generated and applied. Semantic reasoning can be a pivotal tool in this process, enabling the system to understand and interpret the relationships and meanings embedded in diverse data sources such as IoT devices, administrative records, and social media feeds [25].

Semantic reasoning allows the extraction process to identify connections and relationships among disparate data points, providing a coherent and integrated view of various urban phenomena [26]. The employment of machine learning (ML) and data mining techniques further enhances the capability of context extraction. ML algorithms, with their ability to learn and adapt from the data autonomously, and data mining techniques that uncover patterns and connections within the data collectively support the process of turning unrelated data points into significant knowledge.

Moreover, the Semantic Web Rule Language (SWRL) is commonly used in conjunction with ontology languages like OWL for inferencing purposes, serving as an example of rule-based reasoning. The field has also seen the development of various semantic reasoning methods, including SPARQL Inferencing Notation (SPIN), probabilistic reasoning, and ontology-based data access (OBDA). Despite these advancements, a definitive guide or “how-to” is still lacking, with specific methods being employed based on the unique use case and requirements of each project. Tools such as Protégé for ontology management, knowledge graph databases, and the inference engine Apache Jena can be utilized in the process of knowledge extraction.

Current challenges in the field encompass the need for more efficient reasoning algorithms capable of handling large and complex datasets typically found in smart cities. Furthermore, integrating heterogeneous data sources and ensuring the quality and consistency of the extracted knowledge remain significant tasks. Research is ongoing to address these challenges and develop more sophisticated and user-friendly tools and methodologies that can facilitate the process of knowledge extraction and semantic reasoning in various applications, including smart city initiatives.

#### 2.2.4. Inference Application

The final layer in the SeCIS system is dedicated to applying the knowledge generated during the context extraction phase to practical application scenarios. Within this layer, operations like responding to user queries, recommending information aligned with user interests, and similar tasks are executed by leveraging the generated context. The application of this context can take various forms, such as search engines, conversational AI, recommendation systems, question-answering systems, and decision support systems.

For instance, linked open data (LOD) visualization and semantic-based search services exemplify the diverse applications in this layer. IBM’s Watson is a notable example that has developed capabilities to comprehend questions and provide relevant answers, demonstrating the potential and effectiveness of applying semantic knowledge in real-world scenarios [27]. Furthermore, various AI development platforms and libraries are available to support the implementation of knowledge application systems, including Rasa and OpenAI GPT-3 for AI development and LightFM and Surprise for building recommendation systems. Additionally, tools like Elasticsearch and Apache Solr are often utilized for creating advanced search engines that leverage semantic knowledge.

However, in the process of applying context knowledge, several challenges and considerations need attention [28]. The accuracy and reliability of the application’s responses or recommendations are crucial, necessitating rigorous validation and testing processes. Also, ensuring that the system can understand and interpret the user’s intent accurately is vital for providing relevant and satisfactory services. Ongoing research in this area is focused on overcoming these challenges, improving the usability and reliability of inference applications, and exploring new ways of leveraging inferred contextual knowledge to address a broader range of urban issues in smart cities.

### 2.3. Semantic Context Inference System Requirements

Based on the existing literature on semantic context inference, the following summarizes the primary requirements for a SeCIS, categorized into the data access, semantic acquisition, context extraction, and inference application layers:

The fundamental task at the data access layer is providing access to raw data. This necessitates features capable of handling structured, semistructured, and unstructured data, efficient search methodologies, support for large data processing, and functions for data cleaning and preprocessing. The system should also be adaptable to processing various data sources within a smart city, including databases, APIs, web pages, and documents.

In the semantic acquisition layer, there is a need for semiautomated construction technologies capable of transforming collected smart city data into high-quality semantic metadata, alongside strategies for structuring metadata. Given the vast number of data generated within smart cities, it is imperative to derive high-quality semantic metadata that satisfy the requirements for knowledge extraction. Support for ontology mapping and semantic annotation, connecting smart city data to domain ontology concepts, is also essential. While there is ongoing research into powerful NLP features, including named entity recognition (NER), dependency parsing, and coreference resolution, there is a lack of concrete application examples, and continuous research is necessary in order to generate high-quality, meaningful metadata.

The primary objective of the context extraction layer is extracting useful syntactic knowledge from semantically rich data. This includes incorporating semantic reasoning functions as previously discussed. Both machine learning and rule-based technologies can be employed for context extraction in this layer. Additionally, there may be a need to handle uncertainties, manage discrepancies, and support the evolution of knowledge over time.

The inference application layer can be examined from three perspectives:

- *System interactivity:* Consideration for interactivity in the SeCIS is crucial for reflecting the needs of context extraction. An easy-to-use interface allowing for interaction between users or applications and the context base is vital. Such interfaces should not be overly complicated or difficult to manipulate, as this would render them impractical and unusable, necessitating careful consideration.
- *Value-added services:* The system should provide value-added services using the extracted context. This might include advanced search functions, knowledge visualization, intelligent recommendations, and automatic summarization. If these services are too complex, users unfamiliar with the semantic characteristics requiring significant background knowledge may resist using them.
- *Performance evaluation:* This layer should be able to evaluate the performance of the knowledge extraction process and provide feedback for improvement.

The requirements for SeCIS are summarized in Table 1.

## 3. Conceptual Model of the SeCIS

This section presents a conceptual model of SeCIS, designed to incorporate the requirements outlined previously. The SeCIS model is designed to integrate advancements and functionalities in semantics, ensuring robustness and capability to handle semantic context inference tasks in diverse applications.

### 3.1. Proposed Conceptual Architecture

**Figure 1:**
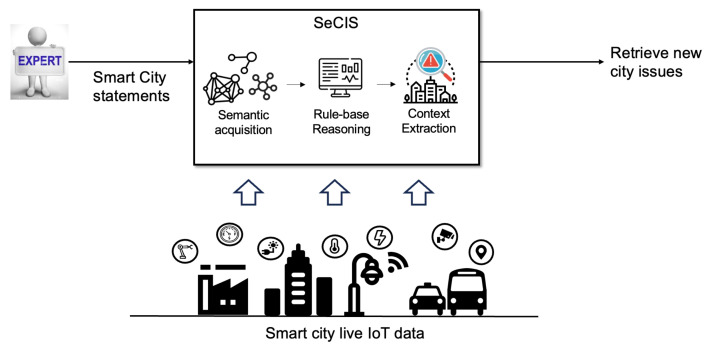
Interaction overview with SeCIS in semantic smart city.

The conceptual model of SeCIS is organized into four layers: the data access layer, semantic acquisition layer, context extraction layer, and inference application layer. Each layer plays a crucial role in transforming raw data into actionable insights. The concept overview of the proposed system is shown in Figure 1.

Upon examining the architecture outlined, we begin with the data access layer, where the target encompasses all data that can be collected within a smart city. This layer addresses the question, “Where is the target (baseline data) from which syntactic knowledge is intended to be extracted?” It achieves this by gathering a vast number of data from various sources, including different IoT platforms, open data portals, public APIs, and legacy platforms.

Next, the semantic acquisition layer targets the extensive data aggregated by the data access layer. Its primary role is to transform raw data into meaningful metadata, providing annotations with semantic information. This process facilitates efficient context extraction and application in subsequent layers. To ensure that the data are interpretable by both humans and machines (software), it is represented using machine-readable languages like RDF and OWL.

The third layer, the context extraction layer, focuses on the semantic data assembled by the semantic acquisition layer. This section is dedicated to solving the problem of “How to extract context?” It accomplishes this by conducting semantic mashups and inference based on specific rules, ultimately deriving results.

Finally, the inference application layer, targeted at smart city residents or administrators, is an active interface where enhanced services, drawn from the extracted semantic context, are provided to users. This interactive layer utilizes the extracted semantic context to offer enhanced services to users, providing an elevated level of utility and value through the efficient application of semantic context in various user-centric services and solutions.

### 3.2. Layered Composition of the Conceptual Model

**Figure 2:**
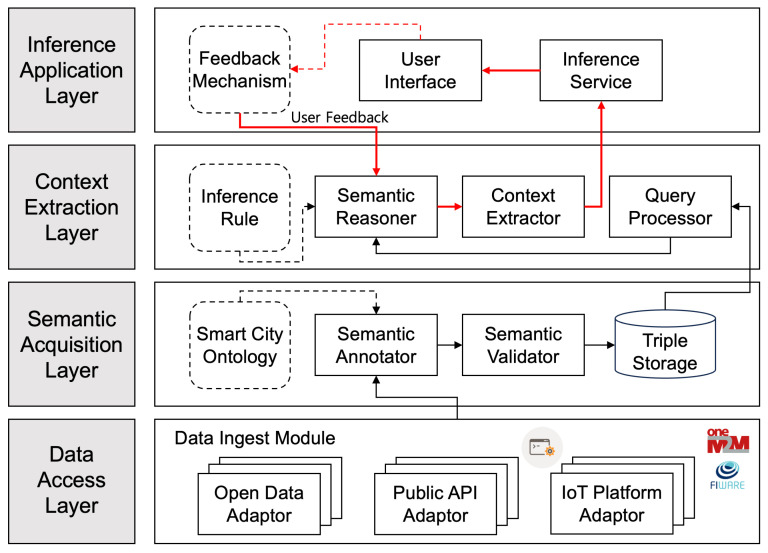
An architecture of conceptual model.

Our proposed SeCIS system is structured into four distinct layers, each with a specialized function that contributes to the system’s ability to understand and infer meaningful context from urban data. Figure 2 is the architecture of the overall conceptual model.

#### 3.2.1. Data Access Layer

At the foundation of SeCIS is the data access layer, which serves as the primary interface between the system and the myriad of data sources present in a smart city ecosystem. This layer is equipped with various adaptors that are tailored to the unique protocols and data formats encountered in urban data environments. The open data adaptor retrieves data from open portals provided by organizations, governmental bodies, and institutions, handling various formats like CSV, XML, and JSON. The public API adaptor, interfacing with public APIs, facilitates access to data offered by service providers, social media platforms, and other organizations, supporting protocols like REST, SOAP, and GraphQL. The IoT platform adaptor, designed for data accumulation from deployed IoT devices, supports connectivity protocols like MQTT, CoAP, and WebSockets, enabling real-time or near-real-time data collection.

The adaptors play a crucial role in not only fetching data from their respective sources but also in normalizing these data into a uniform format suitable for semantic processing. These adaptors, while fetching data, also monitor sources for updates, ensuring access to current data and decoupling the system from data source specifics, enhancing modularity and easing maintenance and expansion tasks.

#### 3.2.2. Semantic Acquisition Layer

Building upon the foundation laid by the data access layer, the semantic acquisition layer is where raw data begin their transformation into a semantically rich representation. The smart city ontology introduces predefined concepts, relationships, and terms representing smart city domains. It establishes a shared vocabulary used for data annotation and integration, articulating complex relationships within smart city data.

The semantic annotator translates raw data into enriched metadata, using natural language processing techniques to identify and establish relationships between data entities, aligning them with ontology concepts. The semantic validator ensures annotated data integrity, consistency, and quality, rectifying errors or inconsistencies against ontology rules. Triple storage, designed for storing data in triple format, supports the efficient querying and retrieval of semantic data.

#### 3.2.3. Context Extraction Layer

The inference rule outlines logic for context inference, identifying patterns and relationships within annotated data. The semantic reasoner applies logic and reasoning to deduce new context, navigating through entity relationships within semantic data. The context extractor identifies and extracts valuable context pieces, transforming them into structured contexts accessible and useful for various applications.

The context extractor plays a significant role in transforming raw semantic data into actionable insights and information that can power a range of smart city applications. Lastly, the query processor is designed to facilitate efficient and flexible querying of the semantic knowledge base. The processor supports various query languages and formats, providing a versatile interface for accessing and retrieving context from the semantic knowledge base. This layer is pivotal, transforming a network of semantic data into a repository of context ready for application.

#### 3.2.4. Inference Application Layer

At the pinnacle of SeCIS is the inference application layer, where the deduced context is translated into actionable insights. The feedback mechanism improves system performance and accuracy by collecting and incorporating user feedback into the system. Through this mechanism, the system gains insights into its performance and areas where it might fall short, facilitating an iterative improvement process. The collected feedback is analyzed and incorporated into the system, refining the extraction algorithms, query processing, and context representation to better align with users’ needs and expectations.

The user interface, designed to be user-friendly and intuitive, enables smooth interaction between users and the system, making semantic context accessible to a broad audience. Lastly, the application interface, serving as a bridge between the extraction system and external applications, provides APIs for seamless external application integration with the knowledge base. Through these APIs, applications can query, retrieve, and utilize the extracted context efficiently, enabling the development and deployment of various smart city services and applications that are powered by the rich semantic context generated by the system. Through this layered approach, SeCIS encapsulates the complexity of smart city data, transforming them into a structured semantic context that serves as the backbone for intelligent urban management and services.

## 4. System Procedure and Technologies of SeCIS

This section describes the implementation of SeCIS. Currently, SeCIS has completed its prototype development. SeCIS undergoes a four-step construction process to provide semantic context extraction services: ontology construction, semantic graph creation, rule-based semantic reasoning, and inference service provision. Firstly, it constructs a smart city common ontology defined by the data hub [13,22]. In the semantic graph creation step, the semantic graph is generated based on ontology templates.

The rule-based semantic reasoning step infers initial knowledge of situations occurring in the city in sentence form, based on geolocation and temporal information, following the 5H1W principles. In the semantic context extraction step, the system displays sentence-like city situations through a user interface and implements a mechanism to derive city issues based on received feedback. The second level of context extracted through user interaction is eventually provided in the inference service.

The provision of inference services can be summarized in a process that includes generating additional metadata from various sources in the smart city, extracting knowledge from the constructed semantic data, and delivering inference services. However, since this study was at the prototype development stage, the targets of inference services, as well as the scope of ontology and metadata, were limited to domains like parking, weather, and air quality. Below, implementation cases for the major functions are presented. Explanations regarding the implemented meanings are also provided in the following sections.

### 4.1. Step 1: Ontology Construction

The development of ontology-based application services commences with the creation of an ontology schema. As a sophisticated knowledge system, the schema stipulates shared concepts within a domain by defining classes, attributes, and relationships, thus providing a structured framework for information representation.

There are substantial advantages to utilizing external ontologies. Firstly, it provides an opportunity to leverage pre-existing knowledge structures, which significantly reduces the time and resources necessary for building ontologies from scratch. This approach also ensures consistency in data representation, fostering effective communication and seamless data exchange across different systems and domains. Furthermore, the adoption of widely recognized external ontologies enhances the system’s interoperability, facilitating a more adaptable and efficient integration process with various data sources and applications.

#### 4.1.1. Justification for Ontology Selection

Selecting the right ontology for a smart city application is critical due to the need for a standardized conceptual framework that allows for interoperability and accurate semantic representation across various systems. The validation of the selected ontology is equally important in order to ensure that it meets the specific requirements of the application. SAREF4City, an extension of SAREF (Smart Appliances REFerence), and SEAS (Smart Energy Aware Systems) ontology are prominent ontologies utilized within the smart city domain [29,30]. However, these ontologies have inherent limitations, such as missing crucial smart city concepts or presenting complexities in adaptability due to intricate relationships between their classes. For instance, certain concepts crucial in the smart city environment, such as the saref:Service class, are not included in SAREF4City. Moreover, when evaluated based on adaptability, complexities arise due to intricate relationships between SEAS’s seas:Property and seas:FeatureOfInterest, which hinder the ontology’s expansion. Due to these limitations, it is difficult to directly use these ontologies to semantically represent various smart city data.

Therefore, we recognize the need to build an ontology that can precisely reflect the characteristics of each subdomain while providing an integrated and consistent semantic expression for the smart city domain. To this end, the ontology construction in our study begins with adopting the common ontology and domain ontology defined by the data hub. The decision to develop a hybrid ontology, combining the common ontology of the data hub with domain-specific ontologies, was driven by the following considerations:

- Coverage: Our analysis indicated that existing ontologies like SAREF4City and SEAS lack comprehensive coverage in critical smart city domains, such as specific services or infrastructural elements.
- Interoperability: We aimed to enhance interoperability by aligning with widely adopted standards, which is a strength of the common ontology structure.
- Modularity and expandability: A modular approach allows for greater flexibility and scalability, accommodating the evolving nature of smart city technologies and applications.

The common ontology functions as a foundational framework, encapsulating standard concepts and relationships applicable across various subdomains within the smart city realm. Upon this foundational structure, domain-specific ontologies are constructed, defining specialized concepts, attributes, and relationships tailored to each domain. This approach provides detailed and nuanced semantic descriptions while considering the complexity and diversity of the smart city domain, thereby supporting modularity and expandability. This deliberate selection process, guided by the necessity for comprehensive coverage and interoperability, leads us to the construction of a hybrid ontology, which we will now discuss in detail.

#### 4.1.2. Competency Questions for Ontology

Competency questions (CQs) serve as a litmus test for validating the ontology’s ability to meet its intended purposes. The CQs devised for our ontology address the representational comprehensiveness, integration capability, and support for the evolution of smart city subdomains. Answering these questions helps confirm the ontology’s practical applicability beyond a theoretical construct. The CQs for our ontology are designed to address the following:

1. Can the ontology represent all the necessary concepts and relationships found in the smart city domain?
2. Does the ontology allow for data integration from heterogeneous sources while maintaining semantic integrity?
3. Is the ontology capable of supporting the evolution and addition of new subdomains within the smart city context?

**Figure 3:**
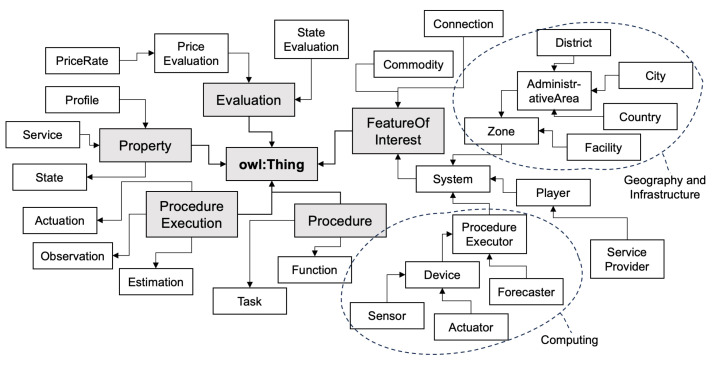
Categorization of system hierarchy with common ontology based on domain scope.

The common ontology of the data hub is structured around six principal high-level concept classes as illustrated in Figure 3. These high-level classes are further delineated into various subclasses, each contributing to the definition of the scopes of these high-level classes. Consequently, this structure results in six distinctive hierarchies, each characterized by unique features, restrictions, and relationships among classes.

Take the high-level class *FeatureOfInterest* as an example. Unlike the SEAS ontology, the characteristics of the class named common:FeatureOfInterest remain constant over time. This class encompasses entities that represent systems, connections, and other related concepts, serving as a foundational framework within the common ontology. Further, the hierarchy of the system can be categorized in various ways under the common:FeatureOfInterest class. Figure 3 showcases two such categorizations as below:

- *Geographical and infrastructural:* This is represented by the subclass common:Zone. The definition of common:Zone in the common ontology is broader compared to its counterpart in the SEAS ontology. It encompasses areas characterized by specific features, purposes, or restrictions, providing a more expansive and inclusive understanding of geographical and infrastructural domains.
- *Computing:* This is represented by the subclass common:ProcedureExecutor. This class is instrumental in representing devices such as sensors and actuators. Notably, common:ProcedureExecutor occupies a higher position in the parent class within the common ontology compared to the SEAS ontology.

Employing a hybrid ontology offers advantages like enhanced semantic precision and domain-specific flexibility. By creating a common ontology that reflects shared concepts across subdomains, our approach ensures that semantic precision is maintained. Domain-specific ontologies allow for the representation of unique and complex relationships, providing a nuanced understanding of each subdomain. Nonetheless, it presents challenges in terms of increased complexity and the need for stringent alignment between the common and domain-specific ontologies.

- Complexity: The hybrid structure may lead to greater complexity in ontology management and necessitates careful integration.
- Alignment challenges: Aligning the common ontology with domain-specific ontologies requires ongoing governance to maintain semantic coherence and prevent discrepancies.

### 4.2. Step 2: Semantic Graph Creation

Data collected from IoT sensors installed in smart cities are usually stored in IoT platforms in row data. For example, data collected from a sense that checks the availability of a particular spot in a parking lot are stored as “1” (available) and “0” (occupied). In this step, we describe the process of converting raw data into triple data by adding additional information and meaning, such as the place, time, and type, and defining the relationship with other data.

#### 4.2.1. Target Data

Related to data sources, the SeCIS system collects data from an array of sources including IoT devices, traffic sensors, and municipal databases. Especially, the target data for semantic data creation primarily pertain to smart city domains, focusing on parking, weather, and air quality as the main areas in this prototype stage. Below is a JSON example of target data representing a parking lot in the smart city data schema:

The JSON-LD serialization of the parking lot entity depicted in Listing 1 is vital as it is modeled in alignment with NGSI-LD standards. NGSI-LD is a specification developed by the ETSI (European Telecommunications Standards Institute) for managing context information in smart city environments. This standard provides a consistent way to describe real-world entities and their relationships, making the data easily understandable and shareable across various applications and services within the smart city data hub. In the data hub, smart city data are modeled via NGSI-LD, ensuring that the structured data are represented semantically, facilitating interoperability and integration with various data sources and applications.

**Listing 1.** Smart city data schema example.1
{
2
   "id": "urn:datahub:ParkingLot:yatap_01",
3
   "type": "ParkingLot",
4
   "name": {
5
      "type": "Property",
6
      "value": "yatap_01"
7
   },
8
   "availableSpotNumber": {
9
      "type": "Property",
10
      "value": 40,
11
      "observedAt": "2021-11-15T20:10:00"
12
   },
13
   "location": {
14
      "type": "GeoProperty",
15
      "value": {
16
        "type": "Point",
17
        "coordinates": [
18
          127.1293735,
19
          37.4114424
20
        ]
21
      }
22
   }
23
}


#### 4.2.2. Annotation Template

The semantic data, also called semantic graph, are built by the combination of triple components that can eventually be linked among multiple graphs. To create this semantic graph, SeCIS uses mapping blueprints, i.e., annotation templates. The template includes descriptors written in human/machine-readable language that describe the annotation characteristics, including the list of required graph representations to be instantiated to create the required semantic graph(s). As multiple domains have verticals with similar or different requirements and characteristics can run on top of a smart city, the AT for each domain is activated via the semantic annotator.

**Figure 4:**
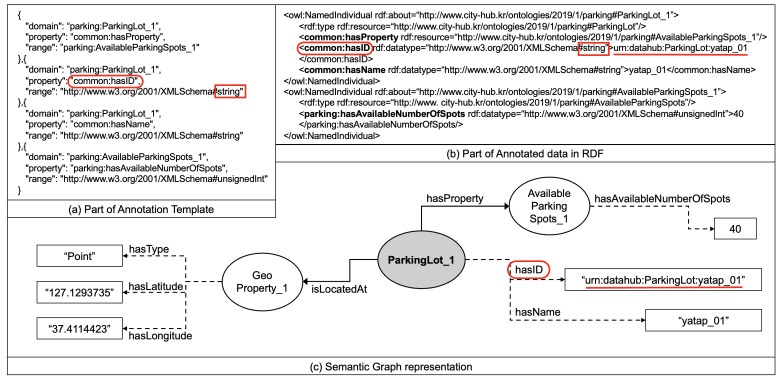
An example in which the smart city data schema is annotated into a semantic graph.

The annotation template as in Figure 4a is created by selecting only the minimum required classes, properties, and relationships for each domain among the annotation rules described in smart city ontologies for annotating the considered nonsemantic data in JSON format. The annotation template structure can be dissected as follows:

- domain: Specifies the subject class of the annotation, acting as the starting point of the relationship or property in the triple. In the given example, it refers to an entity in the parking domain, particularly a specific parking lot denoted as parking:ParkingLot_1.
- property: Indicates the predicate or property of the annotation, establishing a relationship between the domain and range. This component defines what kind of attribute or relationship the domain entity possesses. For example, common:hasProperty signifies that the specified parking lot has a certain property, while common:hasID and common:hasName imply that the parking lot entity has an ID and a name, respectively.
- range: Represents the object class or datatype of the annotation, serving as the target of the relationship or property initiated by the domain. Depending on the property, the range can either be another entity or a datatype. In this case, it could be another entity like parking:AvailableParkingSpots_1 or a datatype as defined in the XML schema, such as a string or unsigned integer.

Each object in the array of the annotation template represents a triple, consisting of a domain, property, and range. Together, these triples help to construct a semantic graph by defining entities (domains), their attributes or relationships (properties), and the characteristics or linked entities of those attributes (ranges).

#### 4.2.3. Semantic Annotation

The central purpose of the annotation is to discern the relationship between ontology and the smart city data schema. Historically, many annotations were manually crafted, making them specific to particular environments and limiting scalability—especially in settings where diverse datatypes, such as those in smart cities, coexist. Within SeCIS, the semantic annotation process constructs a semantic graph utilizing annotation templates. This section elucidates how the smart city data schema, illustrated in Listing  1, becomes annotated into a new semantic graph (as shown in Figure 4c) based on the annotation template presented in Figure 4a.

Examining the smart city data schema, we note that each entity has an ID to distinguish its identity, with the entity type labeled ParkingLot. Now, consider the annotation template in Figure 4a, which is tailored for the parking domain. It reveals a portion of the essential structure required for constructing a semantic graph named parking:ParkingLot_1. For the process of semantic annotation, the ID of an entity is defined as a string datatype through the common:hasID relationship attribute, as highlighted in (b). For a clearer graph representation, (c) illustrates that the semantic graph ParkingLot_1 is connected to a string type of data via the hasID attribute. In this manner, the annotated RDF graph encapsulates the relationships and properties between parking lots and available parking spots, with each entity distinctly defined with its respective attributes, all in accordance with the semantic rules set forth in the annotation template.

The automated annotation process in SeCIS is designed with scalability in mind, allowing the system to accommodate new domains simply by integrating corresponding annotation templates. This modular approach eliminates the need for any changes to the annotation engine itself when expanding to cover additional aspects of smart city data. For instance, if a ”Smart Lighting” domain is to be added, a new template is created and deployed, enabling SeCIS to interpret and annotate ”Smart Lighting” data seamlessly alongside existing domains. This feature underscores the system’s capacity for growth and adaptation, demonstrating its ability to evolve alongside the smart city it serves.

### 4.3. Step 3: Rule-Based Semantic Reasoning

In Step 3, the system derives context that could potentially be the cause of urban issues through rule-based reasoning grounded on the 5W1H principles (who, what, when, where, why, and how). This stage is crucial for generating insights from the data and for further applications and analyses that rely on the context in which the data are used. We considered crucial factors in this process such as time, location, and weather.

#### 4.3.1. Definition of 5W1H Principles

The 5W1H approach is central to SeCIS’s methodology. Below is a detailed walkthrough of its application:

- Who: SeCIS identifies stakeholders or entities involved in any urban issue. This can range from individuals, groups, or organizations. In a traffic context, the Who could pertain to drivers, pedestrians, or traffic control entities.
- When: The system timestamps every situation or event, allowing us to understand its occurrence in real time or its historical context. This aids in determining patterns or anomalies.
- Where: Geospatial data are integrated to pinpoint the exact location of the event. This assists city management in localizing resources or interventions.
- What: This step focuses on determining the nature of the situation or event. Using the traffic scenario, the system might recognize What as a traffic jam, roadwork, or a public event.
- How: This refers to the modality or manner in which the event is happening. In terms of traffic, How could be described by the severity of congestion, the number of lanes affected, or the duration of the disruption.
- Why: This might be the most complex step. The system will attempt to infer the cause or reason behind the event. For the traffic jam scenario, reasons might include an accident, a public event, or roadwork.

For each urban issue, SeCIS aggregates data that align with these categories. Subsequently, this information is integrated using sector-specific semantic reasoning. The merged data, presented as a semantic graph, pave the way for a refined grasp and forecast of urban scenarios. By systematically integrating the 5W1H principles as detailed above, SeCIS provides an organized method to autonomously decipher and tackle urban dynamics. This well-defined strategy guarantees a multifaceted analysis of potential urban issues, amplifying the likelihood of timely detection and effective countermeasures.

#### 4.3.2. Context Modeling Applying 5W1H

Context modeling refers to the systematic representation and structuring of the context within which the data are collected and interpreted. Effective context modeling is imperative for understanding the nuances and implications of the data, as it provides a framework that explicates the relationships, conditions, and parameters that influence the data. In this paper, the 5W1H principles serve as a comprehensive framework guiding the rule-based reasoning process. These principles help in identifying and organizing the essential pieces of information within a context, thereby facilitating a deeper understanding of the situations and issues unfolding within the urban environment. By systematically addressing each principle, the reasoning process holistically considers all relevant facets of the situation, ensuring a thorough and nuanced derivation of context.

Key factors like time, location, and weather play a significant role in the derivation and accumulation of context. The system pays careful attention to these elements, acknowledging their influence on urban dynamics and their contribution to the emergence and resolution of urban issues. By considering these factors, the rule-based reasoning process is grounded in the reality of urban life, ensuring that the derived context is not only relevant but also reflective of the actual conditions and situations in the city.

**Figure 5:**
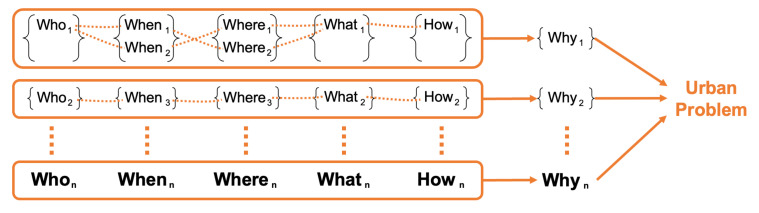
5W1H elements come together to form one *Why*, and multiple *Why*s come together to extract one city problem.

As illustrated in Figure 5, each identified cause (*Why*) of a city problem is associated with one or multiple elements of *Who*, *When*, *Where*, *What*, and *How*. This structure acknowledges the multifaceted nature of urban problems, reflecting the intricate web of events and factors contributing to the emergence of issues in the city landscape. Following is the context formulation of urban events. Each urban event $E_{i}$ can be defined using the 5W1H principles as:

$$E_{i}=\left\{Who_{i},What_{i},When_{i},Where_{i},How_{i}\right\}$$

where *i* is the index of the event. Also, the following is the derivation of *Why* contexts. Each *Why* context is a function of the respective events, formulated as:

$$\begin{array}{cc} Why1 & =f_{1}\left(E_{1},E_{2},\ldots ,E_{n}\right)\\ Why2 & =f_{2}\left(E_{1},E_{2},\ldots ,E_{n}\right)\\ Why3 & =f_{3}\left(E_{1},E_{2},\ldots ,E_{n}\right)\\ Why4 & =f_{4}\left(E_{1},E_{2},\ldots ,E_{n}\right) \end{array}$$

Functions $f_{1},f_{2},f_{3},f_{4}$ represent the relationships between the events and their contribution to each *Why* context.

**Figure 6:**
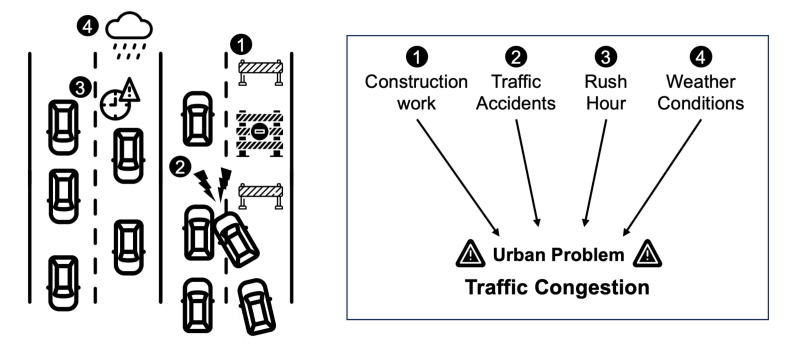
Scenario of urban problem: traffic congestion.

For example, traffic congestion in a city scenario can be explored by understanding how various events (each defined by Who, What, When, Where, and How) culminate to form specific contextual events (Why), and how these, in turn, contribute to a larger urban problem, in this case, traffic congestion as illustrated in Figure 6. Each Why is a significant event context derived from various factors (Who, What, When, Where, How). Below are examples of a few Why contexts in this case:

- Why1: construction-induced congestion.
- Why2: accident-induced congestion.
- Why3: rush-hour congestion.
- Why4: weather-induced congestion.

Table 2 provides a detailed breakdown of factors leading to the identified Why contexts, outlining who is involved, what is happening, when and where these events typically occur, and how these factors contribute to traffic congestion. Understanding these underlying factors is crucial for formulating effective solutions and interventions to address the urban issue of traffic congestion.

As a resulting urban problem, given the derived Why contexts, the urban problem of traffic congestion can be defined as a function of these contexts:

UrbanProblem(TrafficCongestion)=F(Why1,Why2,Why3,Why4)

The function *F* illustrates how the Why contexts collectively contribute to traffic congestion. Understanding this relationship is crucial for developing effective strategies to mitigate congestion and improve urban mobility.

#### 4.3.3. Context Derivation through Rule-Based Reasoning

Semantic reasoning rules, meticulously framed based on the 5W1H principles, are utilized on semantic graphs to extract and interlink essential contexts pivotal for discerning the complex interplay of urban challenges. The formulated rules are crafted employing Semantic Web Rule Language (SWRL)—a powerful language that augments the expressive capacity of OWL ontologies, facilitating the specification of complex rules and relationships between ontology classes and properties.

First, crafting SWRL rules based on the 5W1H principles. The SWRL rules are systematically crafted by translating the 5W1H model into actionable rule components. Each rule embodies the essence of Who, What, When, Where, Why, and How, by associating ontology classes and properties with corresponding 5W1H components. For instance, a rule pertaining to construction-induced congestion would incorporate constructors, road construction events, working hours, specific locations, and the method of construction as constituent elements, thereby creating a comprehensive representation of the contextual event.

For the next step, the designed SWRL rules are subsequently applied to semantic graphs, acting as filters and connectors that sift through and link relevant data points and relationships within the graph. The application process is dynamic and considers various vital elements, including time, location, and prevailing weather conditions, creating a coherent narrative that mirrors the multifaceted and volatile nature of urban landscapes.

To illustrate the application of SWRL rules, let us consider a rule designed to capture the relationship between adverse weather observations and traffic status for the derivation of the “Why4: Weather-Induced Congestion” context. This rule might be formulated as follows:

WeatherObservation(?w) ^ hasObservedValue(?w, "adverse:true") ^

TrafficStatus(?t) ^ hasLocation(?t, ?l) ^ hasLocation(?w, ?l) ^

hasTrafficCondition(?t, "normal") → hasTrafficCondition(?t, "congested")

In this rule:

- WeatherObservation(?w) identifies any weather observation entity ?w.
- hasObservedValue(?w,“adverse:true“) checks if the weather observation is adverse.
- TrafficStatus(?t) identifies any traffic status entity ?t.
- hasLocation(?t,?l) and hasLocation(?w,?l) ensure that the weather observation and traffic status share the same location.
- hasTrafficCondition(?t,“normal") checks if the current traffic condition is normal.
- The rule then infers hasTrafficCondition(?t,“congested") if there is adverse weather at the location of normal traffic status.

Thus, this rule helps in identifying traffic congestion due to adverse weather conditions, contributing to the Why4 context of weather-induced congestion.

#### 4.3.4. Contextual Analysis and Accumulation

The accumulated contexts, derived through rule-based reasoning, provide invaluable insights into potential causes and contributing factors of urban problems, serving as a solid foundation for further analysis and problem solving initiatives within smart cities. As the system continuously receives and processes new data, it dynamically accumulates context derived from the ongoing rule-based reasoning. The accumulation of context is not a mere aggregation but a thoughtful process of connecting and integrating different pieces of context to form a multifaceted understanding of urban issues. This cumulative understanding evolves over time, adapting to changing circumstances and emerging patterns within the city, thereby providing a continuously updated basis for analysis and intervention regarding the urban problems under scrutiny.

### 4.4. Step 4: Inference Service Provision

In Step 4, the system focuses on providing inference services, which are essential for executing various applications effectively within a smart city environment. This phase involves delivering the inferred and reasoned context, obtained from previous steps to applications. This delivery ensures these applications possess the necessary insights and information for making informed decisions and functioning optimally within the urban ecosystem.

## 5. Validation of SeCIS Implementation

Semantic technologies not only aid in the integration and annotation of data within smart city frameworks but also play a crucial role in validating and reasoning over these data. The validation of the SeCIS is conducted internally, focusing on the integrity and accuracy of the annotated semantic graph and the inference results derived from it. This section aims to validate the effectiveness of the SeCIS in a controlled setting, ensuring the system’s readiness for future empirical validation in real-world scenarios. This internal validation will provide insights into the system’s current capabilities and set the stage for future empirical research.

The validation process examines two core components of the SeCIS: the annotated semantic graph and the inference mechanism. The following evaluation criteria are applied:

- Graph integrity: Evaluates the structure of the semantic graph, ensuring that it accurately represents the domain knowledge with all necessary entities and relations.
- Annotation accuracy: Assesses the correctness and relevance of the annotations applied to the graph, ensuring that the data linked to each entity are precise.
- Inference validity: Examines the inferences drawn by the system to determine their logical consistency and alignment with the intended knowledge representation.

Our validation emphasizes the syntax of the assertions and their logical reasoning, ensuring noncontradiction with the ontology’s axioms. This validation process involves class assertion, object property assertion, and data property assertion as described in Table 3.

The annotated semantic graph with proposed SeCIS is a fully adapted mapping by Openlink Virtuoso Server (https://virtuoso.openlinksw.com/, accessed on 24 Novemver 2023). Subjects, predicates, and objects in Virtuoso are stored as a set of graphs via linked triples. For the validation methodology, the SHACL (https://www.w3.org/TR/shacl/, accessed on 24 Novemver 2023) provides a mechanism to define constraints to validate RDF graphs against a set of conditions. These conditions ensure that the data adhere to specified shapes, which can be seen as schema definitions for the data. For SeCIS, the SHACL shapes are defined based on the ontology and the domain-specific requirements of the smart city data model. The validation process particularly focuses on the validation of the syntax of the assertions, as well as the logical rule, which makes sure that these assertions do not contradict the underlying axioms in the ontology.

First, our ontology validation process examines the common ontology of SeCIS, which comprises 48 classes and 49 object properties. We conducted a detailed validation of a subset of these classes to ascertain their conformation to the domain-specific needs of smart cities. The validation entailed an automated SHACL-based assessment, which identified syntax assertions and logical rule compliance, ensuring noncontradiction with the ontology’s axioms. This thorough verification process resulted in the detection and subsequent rectification of a number of errors, reinforcing the semantic graph’s structural soundness.

Secondly, the inference mechanism within SeCIS leverages the validated semantic graphs to conclude the smart city data. The soundness of these inferences is predicated on the underlying logic defined by the ontology and the SHACL shapes. To validate the inferences, the system uses a two-step approach:

1. Logical consistency: The system first ensures that all inferences maintain logical consistency within the bounds of the semantic graph and the ontological rules. This is achieved through automated reasoning tools that can identify logical discrepancies.
2. Contextual relevance: Each inference is then evaluated for its relevance to the specific scenario within the smart city framework. This involves simulating scenarios and verifying that the inferences contribute meaningfully to the resolution of the scenario.

The validity of the inferences is crucial for SeCIS to be an effective tool in smart city management. Therefore, the inferences are not only checked against the semantic graphs but are also subjected to scenario-based validation, which tests their practical applicability. For automated reasoning, SeCIS utilizes tools such as Apache Jena’s inference engine https://jena.apache.org/, accessed on 24 Novemver 2023), which can process the RDF data against the rules defined in the ontology. Scenario simulation is conducted through a custom-built module within SeCIS that models various smart city situations, from traffic management to emergency response, to assess the practicality of the inferences.

The SHACL validation and inference checks were executed across a set of data points representing a typical smart city domain, transportation. The SHACL validation yielded a conformity rate of over 95% for the semantic graph structure and annotations. The inference mechanism maintained a logical consistency rate of 98%, with all tested inferences aligning with the scenarios’ expected outcomes.

The internal validation of SeCIS has demonstrated that the system is robust in its current form, with a high degree of accuracy in its semantic graph annotations and a sound inference mechanism. The validation process ensures that SeCIS is equipped to handle the complexities of smart city data, making it a reliable tool for future empirical studies and real-world applications. As SeCIS evolves, ongoing validation will continue to be an integral part of its development lifecycle, ensuring that it remains effective in the dynamic context of smart city operations.

## 6. Conclusions

This paper delineated a nuanced exploration into the realm of Semantic Context Inference Systems within smart cities, illuminating their theoretical underpinnings and practical applications. In doing so, it underscored the indispensable role of semantic technologies in bolstering the operational intelligence and functionality of urban environments. Focused on smart city data, the study introduced a novel system—SeCIS—skillfully designed to craft semantic graphs through the utilization of well-defined annotation templates. This methodology facilitates a thorough understanding of urban data, revealing the intricacies of city problems and elucidating the events causing them via rule-based reasoning grounded in the 5W1H principles.

The insights garnered from this study are invaluable for practitioners, developers, and policymakers engaged in smart city initiatives. SeCIS emerges as a pivotal reference for the process of semantic data creation and annotation within the realm of smart cities, serving as a dependable blueprint for professionals in the field. Moreover, the approach outlined for rule-based reasoning and context derivation functions as a practical guide, aiding in the extraction of critical insights from urban data. This, in turn, significantly contributes to addressing and resolving pressing urban challenges effectively.

Future research should focus on further optimizing the SeCIS system, with the goal of enhancing its efficiency and accuracy in the domains of semantic annotation and context derivation. It is imperative to explore advanced reasoning algorithms and refine annotation templates, as these are crucial steps toward improvement. Furthermore, there is a pressing need to expand the application spectrum of the inferred semantic context, with future studies investigating its potential impact and utility across a variety of urban domains and services. In conclusion, this study makes a substantial contribution to both the academic and practical dialogues surrounding smart cities. It not only deepens the comprehension of semantic technologies in urban environments but also provides a clear, practical roadmap for their implementation. This guidance is invaluable for both professionals and academics dedicated to the development of smarter, more efficient urban spaces.

## Figures and Tables

**Table 1:** Requirements for Semantic Knowledge Extraction System.

Layer	Requirements
Data access	Access to raw data Efficient search Large data processing Data cleaning and preprocessing Adapt to various data sources
Semantic acquisition	Semiautomated construction Semantic metadata transformation Ontology mapping and annotation Continuous research for metadata
Context extraction	Extract context from semantic data Include semantic reasoning Employ ML or rule-based tech Handle uncertainties and discrepancies Support syntactic knowledge evolution
Inference application	System interactivity Value-added services provision Performance evaluation and feedback

**Table 2:** Factors contributing to different types of congestion.

	Who	What	When	Where	What
Why1	Construction workers, drivers	Road construction	Day hours	Busy streets, intersections	Lane closures
Why2	Drivers, police	Traffic accidents	Random	Highways, intersections	Lane blockages
Why3	Commuters, students	Heavy traffic	Rush hours	Busy roads	High vehicle volume
Why4	Drivers	Adverse weather driving	Bad weather	All roads	Reduced visibility, slippery roads

**Table 3:** Summary of assertion validation types.

Validation Type	Description
Class assertion validation	Checking that each owl:NamedIndividual class assertion includes a class representation available in the ontology.
Object property assertion validation	Ensuring that object properties in assertions are declared in the ontology, along with validating their domain and range involving named individuals.
Data property assertion validation	Verifying that data properties used in assertions are declared in the ontology and that their domain refers to valid named individuals with ranges including valid XSD data values.

## Data Availability

Data are contained within the article.
